# Loss of disease tolerance during *Citrobacter rodentium* infection is associated with impaired epithelial differentiation and hyperactivation of T cell responses

**DOI:** 10.1038/s41598-017-17386-y

**Published:** 2018-01-16

**Authors:** Eugene Kang, Guangyan Zhou, Mitra Yousefi, Romain Cayrol, Jianguo Xia, Samantha Gruenheid

**Affiliations:** 1 Department of Microbiology and Immunology, McGill University, Montreal, Quebec, Canada; 2 McGill University Research Centre on Complex Traits, McGill University, Montreal, Quebec, Canada; 3 Institute of Parasitology, McGill University, Sainte Anne de Bellevue, Quebec, Canada; 4 Department of Pathology and Cellular Biology, University of Montreal, Montreal, Quebec, Canada; 5 Department of Animal Science, McGill University, Sainte Anne de Bellevue, Quebec, Canada

## Abstract

*Citrobacter rodentium* is an intestinal mouse pathogen widely used as a model to study the mucosal response to infection. Inbred mouse strains suffer one of two fates following infection: self-limiting colitis or fatal diarrheal disease. We previously reported that *Rspo2* is a major genetic determinant of the outcome of *C*. *rodentium* infection; *Rspo2* induction during infection of susceptible mice leads to loss of intestinal function and mortality. *Rspo2* induction does not impact bacterial colonization, but rather, impedes the ability of the host to tolerate *C*. *rodentium* infection. Here, we performed deep RNA sequencing and systematically analyzed the global gene expression profiles of *C*. *rodentium*-infected colon tissues from susceptible and resistant congenic mice strains to determine the common responses to infection and the *Rspo2*-mediated dysfunction pathway signatures associated with loss of disease tolerance. Our results highlight changes in metabolism, tissue remodeling, and host defence as common responses to infection. Conversely, increased Wnt and stem cell signatures, loss of epithelial differentiation, and exaggerated CD4^+^ T cell activation through increased antigen processing and presentation were specifically associated with the response to infection in susceptible mice. These data provide insights into the molecular mechanisms underlying intestinal dysfunction and disease tolerance during *C*. *rodentium* infection.

## Introduction

*Citrobacter rodentium* is a natural mouse pathogen that infects the large intestine and causes colitis and characteristic thickening of the colonic mucosa^1^. It shares several pathogenic mechanisms with human enteropathogenic and enterohaemorrhagic *E*. *coli* and is widely recognized as an excellent model for studying the intestinal response to enteric pathogens^1^. Importantly, disease severity can range from self-limiting colitis to lethal diarrhea and inflammation depending on the genetic background of the host^2,3^.

We previously discovered a novel pathway in susceptible mice (e.g. C3H/HeOuJ, AKR, FVB) that leads to the development of fatal diarrheal disease during *C*. *rodentium* infection through *Rspo2*-mediated disruption of intestinal homeostasis^3^. *Rspo2* encodes a member of the R-spondin family of secreted proteins, which have recently emerged as potent enhancers of the canonical Wnt signaling pathway^4^. This pathway plays a crucial role in regulating epithelial cell fate and determination, and is the driving force behind the proliferation of intestinal epithelial precursors^5^. *Rspo2* is robustly induced during infection in susceptible mouse strains, leading to pathological activation of Wnt signaling, generation of a poorly differentiated colonic epithelium, and animal death^3,6–8^. In contrast, resistant mice that do not express *Rspo2* following infection still develop colonic epithelial hyperplasia at the peak of infection but suffer milder, self-limiting disease without experiencing a loss of intestinal function^3^.

To avoid any confounding genetic and phenotypic differences between divergent inbred strains, we developed a congenic mouse strain that is on a pure C3H/HeOuJ (henceforth called C3Ou) susceptible background but carries a segment of chromosome 15 encompassing *Rspo2* and its regulatory region from resistant C57BL/6 mice^6^. These resistant congenic mice exhibit complete survival following *C*. *rodentium* infection compared to susceptible C3Ou mice which suffer 100% mortality. Furthermore, we previously demonstrated that bacterial loads and infection kinetics are identical in susceptible C3Ou and resistant congenic mice^6^, a phenomenon that is not observed when comparing different susceptible and resistant inbred strains^9^. This indicates that *Rspo2* does not affect bacterial colonization or replication but rather the ability of the host to establish disease tolerance in the presence of pathogenic bacteria in the intestine. Disease tolerance is a host defence strategy that reduces the negative effects of infection on the host without affecting pathogen burden^10^. Our unique parental and congenic strains, differing only in their expression of *Rspo2* during infection, therefore provide a more accurate experimental model system to study the biological effects of *Rspo2* on disease tolerance as compared to those based on genetically divergent inbred mouse strains.

In the present study, we employed RNA sequencing technology to characterize the global shared response to *C*. *rodentium* infection in both susceptible and resistant congenic mice, and to define the complete *Rspo2*-mediated intestinal dysfunction pathway signatures and networks of interacting genes that mediate loss of disease tolerance in susceptible mice. To reduce bias and minimize false positives in our differential expression analyses, we applied two well-established statistical methods to identify a robust list of differentially expressed genes (DEGs) that were further incorporated into a high-quality protein-protein interaction network to create network-based gene signatures. Our work provides an unbiased, global perspective of the common and differential host response to infection and provides new insights into the underlying mechanisms regulating intestinal homeostasis versus dysfunction.

## Results

### Common gene expression patterns associated with *C*. *rodentium* infection

**Figure 1:**
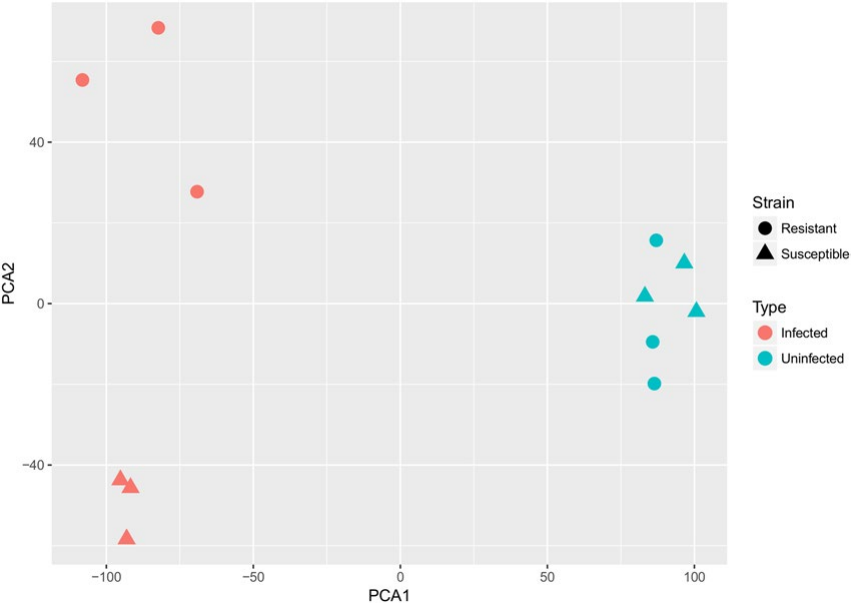
Principal component analysis (PCA) scatter plot reveals separate clustering based on mouse strain and infection status. Colons from three mice per group were harvested at 0 and 9 days post-infection for RNA sequencing and PCA of normalized host gene counts for all samples was generated.

RNA-seq was performed on the Illumina HiSeq. 2000/2500 sequencer to explore the dynamic and global transcriptome profiles of susceptible and resistant congenic mice colons that were either uninfected or infected with *C*. *rodentium* for 9 days, the latest time point in which infected susceptible mice are consistently viable. Consistent with our previous work, we confirmed that bacterial loads were identical in our susceptible and resistant congenic mice at 9 days post-infection (Supplementary Fig. S1). We subsequently performed principal component analysis (PCA) to evaluate the level of similarity in the gene expression patterns of each sample. PCA revealed three distinct clusters: uninfected controls, infected susceptible mice, and infected resistant mice, indicating that samples are closely grouped according to mouse strain and infection status, and that few transcriptomic differences are observed between strains prior to infection (Fig. 1).

**Figure 2:**
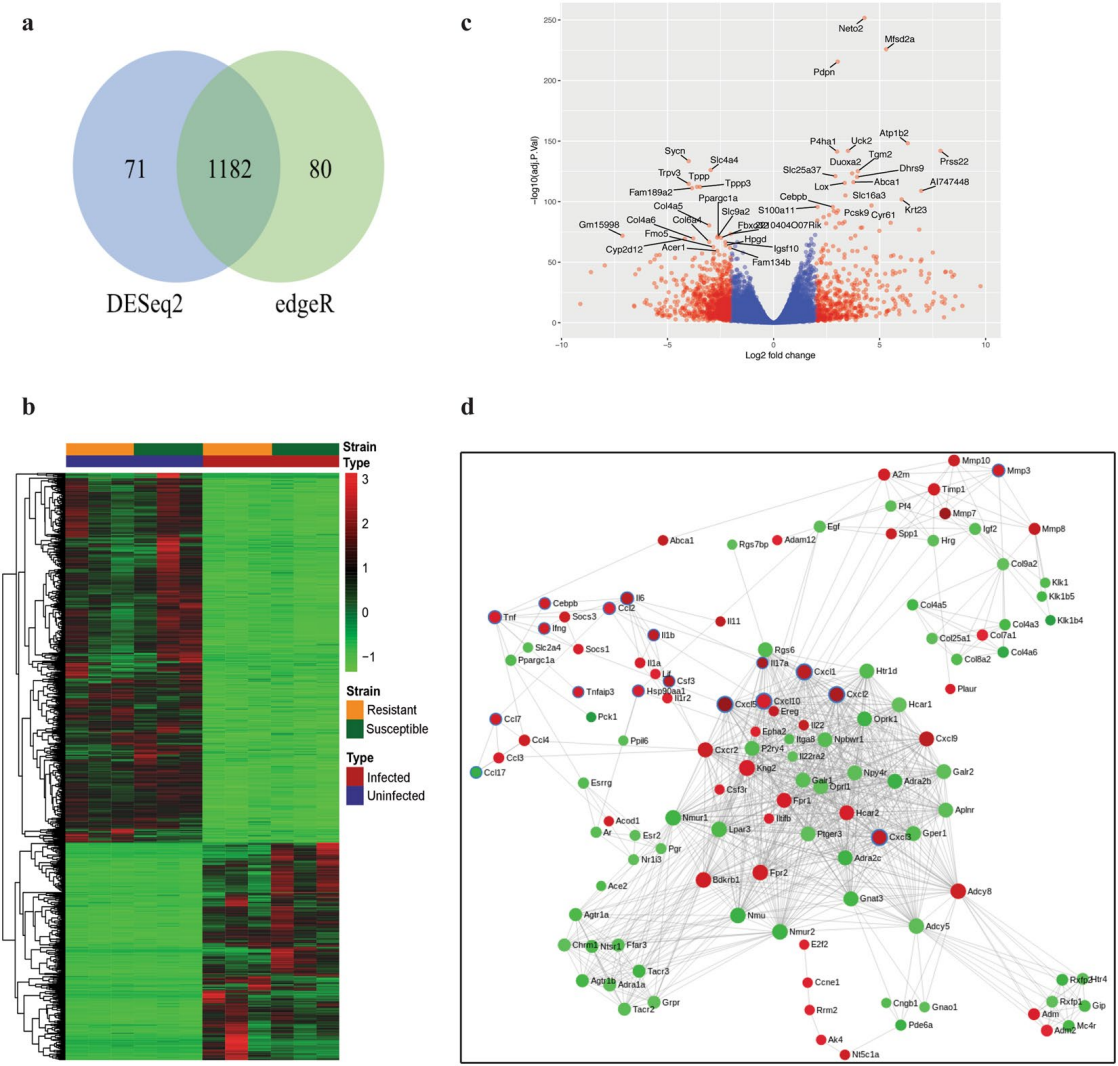
Global overview of the shared colonic response to *C*. *rodentium* infection in susceptible and resistant congenic mice. (**a**) Venn diagram of the overlap between the set of differentially expressed genes (DEGs) found by edgeR and DESeq2. DEGs are those exhibiting a log_2_ fold change of more than 2 and a p-value of less than 0.05. (**b**) Heatmap of normalized read counts of the 1182 DEGs as identified by both edgeR and DESeq2. (**c**) Volcano plot showing all expressed transcripts. DEGs are plotted in red and genes that are not classified as differentially expressed are plotted in blue. Labeled genes are those within the top 20 up- and down-regulated DEG list based on edgeR. (**d**) Largest protein-protein interaction network generated by STRING. Red nodes represent up-regulated proteins and green nodes represent down-regulated proteins relative to steady state. Proteins within the IL-17 pathway are highlighted with blue borders. Solid lines indicate interacting partners.

We first assessed the shared global response to infection in both strains by performing differential expression analysis between the uninfected and infected groups using two well-established methods: edgeR^11^ and DESeq2^12^. Applying a threshold of log_2_ fold change = 2 and adjusted *p* < 0.05, edgeR identified 1,262 DEGs while DESeq2 identified 1,253 DEGs, with a large overlap of 88.7% of DEGs common to both methods (Fig. 2A). A heatmap and volcano plot was generated to visualize the general expression pattern of the transcripts during *C*. *rodentium* infection (Fig. 2B and C). Notably, the number of significantly down-regulated genes was larger than the number of up-regulated genes: 744 genes down-regulated versus 438 genes up-regulated.

#### Top 20 differentially expressed genes

Table 1 lists the top 20 up-regulated and top 20 down-regulated DEGs during infection in both susceptible and resistant congenic mice as measured by edgeR. With regard to the top 20 up-regulated DEGs, many encode products with diverse functions, ranging from those involved in molecule or ion transport (*Abca1*, *AI747448*, *Atp1b2*, *Duoxa2*, *Mfsd2a*, *Slc16a3*, *Slc25a37*), collagen fibril organization (*Lox*, *P4ha1*), cell-cell adhesion (*Pdpn*, *Cyr61*), to cholesterol metabolic process (*Abca1*, *Pcsk9*). Importantly, our results are consistent with previous microarray data of *C*. *rodentium*-infected resistant C57BL/6 mice identifying a number of significantly induced genes that are within our top 20 up-regulated DEG list including *AI747448*, *Atp1b2*, *Mfsd2*, *Cyr61*, *Pdpn*, *Neto2*, *Cebpb*, *Duoxa2*, and *Tgm2*^13^. These data highlight the commonality of this response between susceptible and resistant mice and suggest it may be relevant to all mouse strains, independent of *Rspo2* expression during infection.

**Table 1:** Top 20 up-regulated and down-regulated differentially expressed genes in infected susceptible and resistant congenic mice based on edgeR.

Top 20 Up-regulated Genes			Top 20 Down-regulated Genes		
Symbol	P-value	Name	Symbol	P-value	Name
Neto2	2.05E-252	neuropilin and tolloid-like 2	Sycn	3.16E-134	syncollin
Mfsd2a	1.44E-226	major facilitator superfamily domain containing 2 A	Slc4a4	8.37E-127	solute carrier family 4, member 4
Pdpn	2.31E-216	podoplanin	Trpv3	2.00E-115	transient receptor potential cation channel, subfamily V, member 3
Atp1b2	5.42E-149	ATPase, Na+/K+ transporting, beta 2 polypeptide	Tppp	5.89E-113	tubulin polymerization promoting protein
Uck2	1.03E-142	uridine-cytidine kinase 2	Tppp3	6.43E-113	tubulin polymerization-promoting protein family member 3
Prss22	1.03E-142	protease, serine, 22	Fam189a2	6.83E-112	family with sequence similarity 189, member A2
P4ha1	4.59E-142	procollagen-proline, proline 4-hydroxylase, alpha 1	Col4a5	3.95E-81	collagen, type IV, alpha 5
Tgm2	7.22E-126	transglutaminase 2, C polypeptide	2210404O07Rik	4.74E-74	RIKEN cDNA 2210404O07 gene
Duoxa2	4.30E-124	dual oxidase maturation factor 2	Gm15998	1.52E-72	predicted gene 15998
Slc25a37	8.76E-122	solute carrier family 25, member 37	Ppargc1a	2.32E-72	peroxisome proliferative activated receptor, gamma, coactivator 1 alpha
Dhrs9	3.79E-121	dehydrogenase/reductase (SDR family) member 9	Slc9a2	3.80E-71	solute carrier family 9, member 2
Abca1	7.78E-117	ATP-binding cassette, sub-family A (ABC1), member 1	Fbxo32	6.91E-71	F-box protein 32
Lox	3.56E-116	lysyl oxidase	Col4a6	2.49E-70	collagen, type IV, alpha 6
AI747448	1.25E-109	expressed sequence AI747448	Cyp2d12	6.57E-70	cytochrome P450, family 2, subfamily d, polypeptide 12
Slc16a3	7.48E-106	solute carrier family 16, member 3	Col6a4	1.78E-67	collagen, type VI, alpha 4
Krt23	1.42E-102	keratin 23	Igsf10	2.17E-67	immunoglobulin superfamily, member 10
Cyr61	1.51E-97	cysteine rich protein 61	Hpgd	1.29E-64	hydroxyprostaglandin dehydrogenase 15 (NAD)
S100a11	2.13E-96	S100 calcium binding protein A11 (calgizzarin)	Fmo5	2.43E-63	flavin containing monooxygenase 5
Cebpb	2.35E-96	CCAAT/enhancer binding protein (C/EBP), beta	Fam134b	1.32E-62	family with sequence similarity 134, member B
Pcsk9	2.52E-93	proprotein convertase subtilisin/kexin type 9	Acer1	4.41E-60	alkaline ceramidase 1

Top 20 tables based on DESeq2 are available in Supplementary Table S5.

*C*. *rodentium* infection results in crypt elongation and an expansion in undifferentiated proliferating cells as an epithelial repair mechanism^1,14^. While differentiated cells in the upper crypt utilize butyrate as their primary energy source, undifferentiated proliferating cells exhibit a metabolic reprogramming event often referred to as the Warburg effect by fermenting glucose to lactate^15,16^. *Slc16a3* (also known as *Mct-4*) is a monocarboxylate transporter that shuttles lactate across the cell membrane and is involved in this glycolytic metabolic pathway^17,18^. *Ldha*, another key player in this pathway involved in mediating pyruvate conversion to lactate^19^ was also found to be significantly increased in both strains during infection (*p* = 8.48 × 10^−7^). The highly significant modulation of these genes and in other genes related to channels/transporters such as *Abca1* and *Slc25a37* may be indicative of a metabolic shift in the host epithelium due to infection with *C*. *rodentium*.

Genes associated with collagen fibril organization and cell-cell adhesion are typically involved in extracellular matrix (ECM) remodeling and in wound-healing processes. Indeed, the matricellular protein *Cyr61* has been shown to induce IL-6 expression in macrophages and in fibroblasts during DSS colitis to promote intestinal epithelial cell proliferation and recovery^20^. But other genes including *Lox* and *P4ha1* have collagen-modifying activities and the ability to alter the contents of the ECM through collagen deposition, a phenomenon frequently observed in cancers^21^. The recruitment of stromal cells such as fibroblasts often precedes collagen deposition^21^. Indeed, the increase in *Pdpn* expression, a marker of lymphatic stromal cells in the intestinal lamina propria may reflect the increased migration or presence of stromal cells during infection; since our RNA-seq experiment was performed on whole colon tissues, apparent gene expression changes could either reflect differences in cell populations or differences in gene expression within populations. Along with those already mentioned, other top up-regulated DEGs such as *Uck2*, *Krt23*, *Pcsk9*, and *S100a11* have also been associated with cancer or inflammatory bowel disease (IBD), likely reflecting their roles in cell proliferation or repair^22–25^.

In contrast, genes associated with ECM organization (*Col4a5*, *Col4a6*, *Col6a4*), microtubule polymerization formation (*Tppp*, *Tppp3*), and mitochondrial biogenesis (*Ppargc1a*) were found to be among the top down-regulated DEGs during infection. *Ppargc1a* (also known as *Pgc1α*) is a transcriptional regulator of mitochondrial biogenesis and oxidative phosphorylation in which its reduced expression in cancers including colorectal cancer (CRC) has previously been suggested to contribute to the Warburg effect^26,27^. *Col4a5* and *Col4a6* are type IV collagens and major components of the basement membrane. Notably, transcriptional loss of *Col4a5* and *Col4a6* in the epithelial basement membrane was observed in CRC and during cancer cell invasion^28^. *Tppp* and *Tppp3* are tubulin polymerization-promoting proteins that act to stabilize the microtubular network through positive regulation of microtubule assembly. Collectively, the down-regulation of these genes seems to implicate defects in barrier or structural integrity in the development of colitis. Other genes within our top down-regulated DEG list including *Sycn*, *Trpv3*, *Hpgd*, and *Fam134b* are also found down-regulated in DSS colitis or CRC, suggesting that their decreased expression is a common feature during intestinal tissue damage and inflammation.

#### Significantly enriched functions in global response to infection

Enrichment analysis based on KEGG pathways and gene ontology (GO) terms was applied to examine the biological roles of the entire set of modulated DEGs, while the STRING database was used to highlight important protein-protein interaction networks. To minimize false positives, only genes that were identified using both edgeR and DESeq2 were considered for functional enrichment analysis and generation of the protein-protein interaction networks. KEGG results revealed the IL-17 signaling pathway, cytokine-cytokine receptor interactions, and the TNF signaling pathway to be significantly over-represented, indicating a general defence response to enteric infection in both strains (Supplementary Table S1). As depicted in Fig. 2D, the largest network map generated by STRING and subsequent enrichment analysis on this network highlights the IL-17 pathway and its connections with chemokines, interferons, interleukins, and other immunoregulatory cytokines in infected mice. Importantly, the IL-17 pathway has been demonstrated to be central to the immune response to *C*. *rodentium* infection^29^. Indeed, genes within the IL-17 pathway including *Cebpb*, *Tnf*, *IL-6*, *Cxcl1*, *Cxcl2*, *Cxcl3*, *Cxcl5*, *Cxcl10*, and *Ccl7* were shown to be up-regulated in both susceptible and resistant mice during infection. This data is consistent with a previous microarray study^13^ examining the host defence response to *C*. *rodentium* and suggests that there is no impairment in terms of activation of the immune response in susceptible mice. Other over-represented pathways were involved in omega-6 fatty acid metabolism, as well as in neuroactive ligand-receptor interaction and in serotonergic synapse. Notably, products of arachidonic acid metabolism play a broad role in the regulation of inflammatory responses by exerting numerous pro-inflammatory effects such as in neutrophil migration and promotion of the Th17 pathway^30^. Phospholipases such as *Pla2g2a*, *Pla2g2e*, and *Pla2g4c* which regulate the release of arachidonic acid from cell membranes were indeed found to be up-regulated in both susceptible and resistant congenic mice during *C*. *rodentium* infection (log_2_ fold change of 3.87, 3.11, 3.26, respectively). GO term enrichment analysis revealed patterns similar to KEGG pathway terms (Supplementary Table S2). Overall, our data highlights changes in host metabolism, tissue remodeling through ECM alterations, epithelial proliferation, and immune responses that are common in both genetically susceptible and resistant mouse strains in response to *C*. *rodentium* infection.

### Strain-specific gene expression patterns in *C*. *rodentium* infection

**Figure 3:**
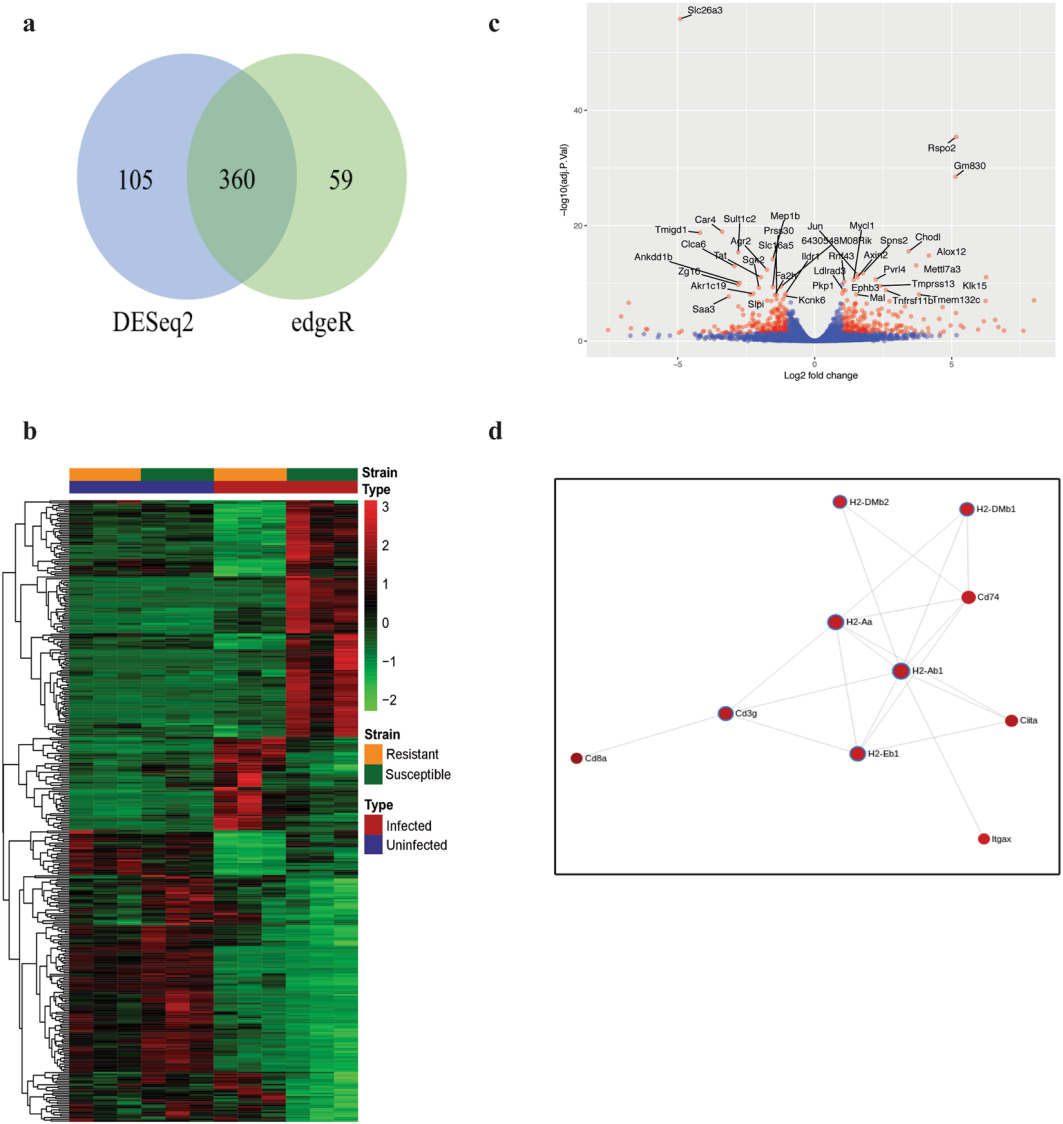
Global overview of the strain-specific colonic response to *C*. *rodentium* infection. (**a**) Venn diagram of the overlap between the set of differentially expressed genes (DEGs) found by edgeR and DESeq2. DEGs are those exhibiting a log_2_ fold change of more than 1 and a p-value of less than 0.05. (**b**) Heatmap of normalized read counts of the 360 DEGs as identified by both edgeR and DESeq2. (**c**) Volcano plot showing all expressed transcripts. DEGs are plotted in red and genes that are not classified as differentially expressed are plotted in blue. Labeled genes are those within the top 20 up- and down-regulated DEG list based on edgeR with the exception of *Ang4* due to it being out of scale. (**d**) Largest protein-protein interaction network generated by STRING. Red nodes represent up-regulated proteins in infected susceptible mice relative to infected resistant congenic mice. Proteins within the Th17 cell differentiation pathway are highlighted in blue and are up-regulated in infected susceptible mice compared to resistant congenic mice. Solid lines indicate interacting partners.

We next assessed strain-specific gene expression patterns following *C*. *rodentium* infection by performing differential expression analysis again using edgeR and DESeq2. Applying a threshold of log_2_ fold change = 1 and adjusted *p* < 0.05, edgeR and DESeq2 identified 419 and 465 DEGs, respectively. As shown in the Venn diagram, this corresponded to a 68.7% overlap of DEGs common to both methods (Fig. 3A). We next generated a heatmap from these 360 overlapping genes to visualize and compare the differential response to *C*. *rodentium* infection in the two strains (Fig. 3B). Consistent with the PCA in Fig. 1, while the gene expression profiles were highly similar in both strains at steady state, there was a substantial difference in response to infection in a subset of genes with 137 genes up-regulated in susceptible mice compared to resistant congenic mice and 54 genes up-regulated in resistant congenic mice compared to susceptible mice.

#### Top 20 differentially expressed genes and GSEA

We previously identified the *Rspo2* gene to control the outcome to *C*. *rodentium* infection in mice; it localizes to the minimal genetic region governing infection outcome and is induced to high levels specifically in susceptible mice^3^. In addition, we previously characterized the colonic transcriptional response of susceptible and resistant congenic mice for a limited panel of specific genes and found genes expressed in differentiated enterocytes *(Slc26a3* and *Car4)* to be dramatically down-regulated in infected susceptible mice^3^, consistent with past studies^31,32^. Differential expression analysis of our dataset as illustrated in the volcano plot (Fig. 3C) and in Table 2 confirmed *Rspo2*, *Slc26a3*, and *Car4* as top DEGs.

**Table 2:** Top 20 up-regulated and down-regulated differentially expressed genes in infected susceptible mice compared to infected resistant congenic mice based on edgeR.

Top 20 Up-regulated Genes			Top 20 Down-regulated Genes		
Symbol	P-value	Name	Symbol	P-value	Name
Rspo2	4.09E-36	R-spondin 2 homolog (Xenopus laevis)	Slc26a3	1.49E-56	solute carrier family 26, member 3
Gm830	3.29E-29	predicted gene 830	Car4	1.12E-19	carbonic anhydrase 4
Chodl	2.93E-16	chondrolectin	Tmigd1	1.70E-19	transmembrane and immunoglobulin domain containing 1
Ang4	5.39E-16	angiogenin, ribonuclease A family, member 4	Sult1c2	4.22E-16	sulfotransferase family, cytosolic, 1 C, member 2
Alox12	1.54E-15	arachidonate 12-lipoxygenase	Mep1b	6.80E-15	meprin 1 beta
Mettl7a3	7.56E-14	methyltransferase like 7A3	Clca6	1.03E-13	chloride channel calcium activated 6
Spns2	1.80E-12	spinster homolog 2	Agr2	4.33E-13	anterior gradient 2
Jun	2.90E-12	Jun oncogene	Tat	8.48E-12	tyrosine aminotransferase
Klk15	8.48E-12	kallikrein related-peptidase 15	Ankdd1b	8.03E-11	ankyrin repeat and death domain containing 1B
Axin2	8.74E-12	axin2	Zg16	1.82E-10	zymogen granule protein 16
Pvrl4	2.14E-11	poliovirus receptor-related 4	6430548M08Rik	3.90E-10	RIKEN cDNA 6430548M08 gene
Mycl1	2.47E-11	v-myc myelocytomatosis viral oncogene homolog 1, lung carcinoma derived	Prss30	4.43E-10	protease, serine, 30
Rnf43	4.88E-11	ring finger protein 43	Sgk2	5.54E-10	serum/glucocorticoid regulated kinase 2
Tmprss13	2.74E-10	transmembrane protease, serine 13	Ildr1	6.25E-09	immunoglobulin-like domain containing receptor 1
Tnfrsf11b	1.50E-09	tumor necrosis factor receptor superfamily, member 11b	Akr1c19	6.25E-09	aldo-keto reductase family 1, member C19
Ldlrad3	1.52E-09	low density lipoprotein receptor class A domain containing 3	Slpi	8.39E-09	secretory leukocyte peptidase inhibitor
Ephb3	1.75E-09	Eph receptor B3	Slc16a5	9.59E-09	solute carrier family 16, member 5
Pkp1	6.25E-09	plakophilin 1	Fa2h	1.12E-08	fatty acid 2-hydroxylase
Mal	8.39E-09	myelin and lymphocyte protein, T cell differentiation protein	Kcnk6	1.53E-08	potassium inwardly-rectifying channel, subfamily K, member 6
Tmem132c	8.99E-09	transmembrane protein 132 C	Saa3	1.95E-08	serum amyloid A 3

Top 20 tables based on DESeq2 are available in Supplementary Table S6.

**Figure 4:**
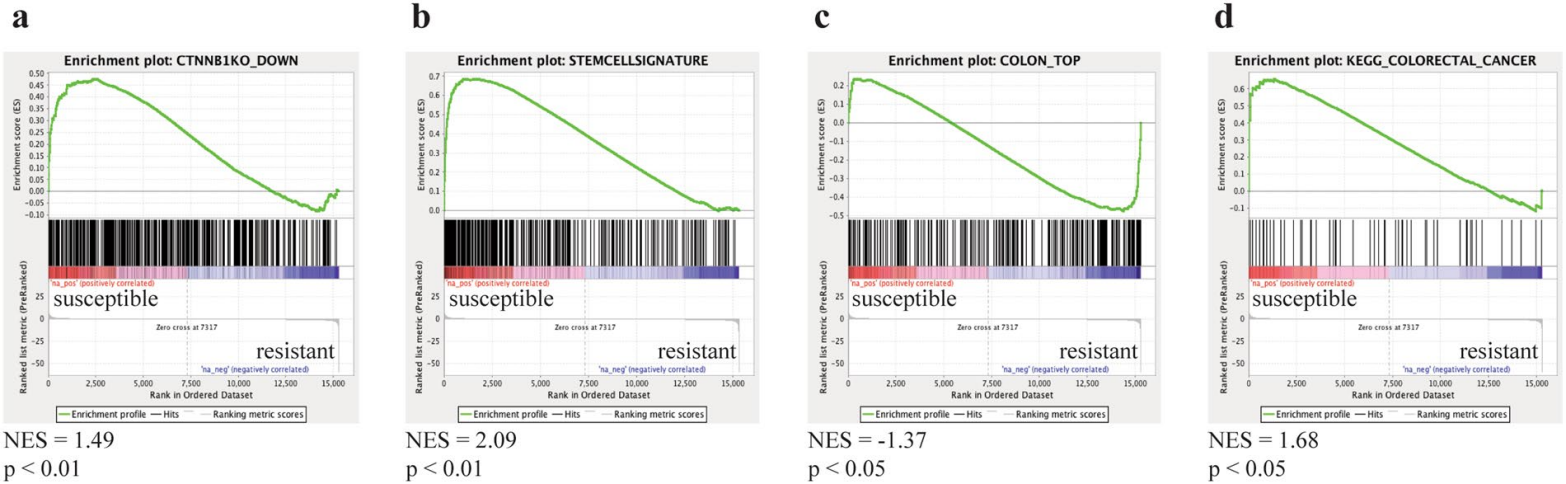
Gene set enrichment analysis (GSEA) reveals correlation between *Rspo2* expression and Wnt target genes, intestinal epithelial cells, and colorectal cancer-related genes. GSEA probing for enrichment of (**a**) β-catenin knockout-associated, (**b**) stem cell-associated, (**c**) colon top-associated, and (**d**) colorectal cancer-associated gene set signatures in infected susceptible and resistant congenic mice. NES = normalized enrichment score.

In our previous work, we proposed that *Rspo2* induction in infected susceptible mice leads to pathological activation of Wnt/β-catenin signaling, induction of intestinal epithelial proliferation, and generation of a poorly differentiated epithelium^3^. Gene set enrichment analysis (GSEA) was conducted as a means to further investigate this hypothesis. Our RNA-seq dataset was correlated to the β-catenin knockout gene set signature^33^ and to the Lgr5^+^ stem cell signature^34^. Consistent with our *C*. *rodentium* susceptibility model of hyperactivation of Wnt signaling, we found significant enrichment of Wnt target genes (Fig. 4A) and in the stem cell signature (Fig. 4B) in infected susceptible mice due to *Rspo2* up-regulation. Furthermore, GSEA correlating our dataset to a gene list obtained by characterizing the gene expression profiles of the top and basal crypt compartments of the human colon^35^ revealed enrichment of genes differentially expressed in the colon top in infected resistant congenic mice compared to susceptible mice (Fig. 4C), illustrating the poorly differentiated state of the epithelium in infected susceptible mice.

Constitutive activation of Wnt signaling constitutes the primary transforming event in CRC^36^. Notably, activating *RSPO2* gene fusions have been identified as being associated with CRC development in humans^37^. In this respect, colon cancer-related genes as well as Wnt target genes (*Jun*, *Axin2*, *Pvrl4*, *Rnf43*, *Tnfrsf11b*, *Ephb3*) were among the top up-regulated DEGs in susceptible mice following infection while genes highly expressed in differentiated cells and genes negatively correlated with CRC or IBD (*Slc26a3*, *Car4*, *Tmigd1*, *Mep1b*, *Clca6*, *Agr2*, *Zg16*) were found to be among the top down-regulated DEGs (Table 2). GSEA correlating our dataset to the CRC gene set signature from the KEGG database confirmed enrichment of CRC-related genes in infected susceptible mice compared to resistant congenic mice (Fig. 4D). These data suggest a new mechanistic link between enteric infection and cancer through *Rspo2*-mediated signaling.

#### Functional enrichment analysis of strain-specific responses to infection

**Figure 5:**
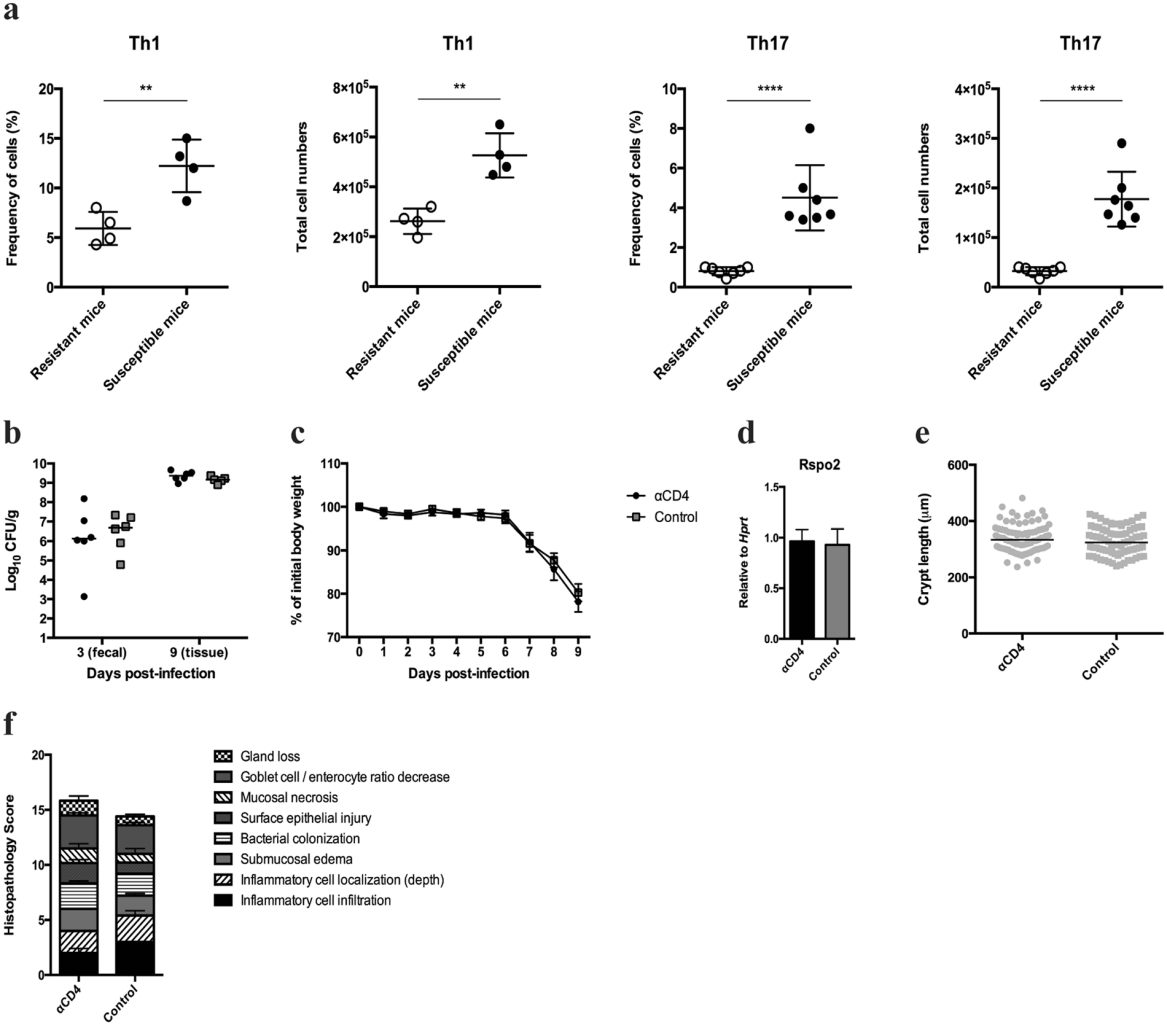
Infection with *C*. *rodentium* results in an amplified Th1 and Th17 cell response in susceptible mice compared to resistant congenic mice but does not contribute to immunopathology. (**a**) Flow cytometry analysis of the frequency and total numbers of Tnf-α^+^ CD4^+^ Th1 cells and IL-17^+^/IL-22^+^ CD4^+^ Th17 cells in the colonic lamina propria of susceptible and resistant congenic mice at 9 days post-infection (n = 4 per group for Th1 data, n = 7 per group for Th17 data; Th17 data is an aggregate of two independent experiments). Data are presented as mean ± SD. **p < 0.01, ****p < 0.0001 by unpaired two-tailed Student’s t test. (**b**–**f**) Susceptible mice were administered 200 μg of either a CD4-depleting antibody or an isotype control intraperitoneally on the day of oral infection with *C*. *rodentium* and again on day 2 post-infection (n = 5–6 per group). (**b**) Fecal and colon tissue CFU was quantified on day 3 and 9 post-infection, respectively. Horizontal bars indicate median values. (**c**) Body weight loss was measured on a daily basis. Data are presented as mean ± SEM. (**d)** Expression of *Rspo2* by qRT-PCR was normalized to *Hprt*. Data are presented as mean ± SEM. (**e**) Crypt measurements were taken from H&E-stained colonic sections. The graph shows measurements of individual crypt lengths. Horizontal bars indicate median values. (**f**) Tissue pathology scores from H&E-stained colonic sections. Data are presented as mean ± SEM. Data was analyzed using the Mann-Whitney test.

Functional enrichment analysis was performed using the KEGG database and GO terms to identify other pathways and biological processes that were affected differentially in susceptible versus resistant congenic mice following *C*. *rodentium* infection. Genes and processes enriched in IBD, cell adhesion molecules, Th1/Th2/Th17 cell differentiation, and antigen processing and presentation were over-represented in infected susceptible mice compared to resistant congenic mice (Supplementary Table S3). Notably, a number of H-2 major histocompatibility complex (MHC) genes were associated with the majority of these pathways: *H2-Ab1*, *H2-Aa*, *H2-DMb1*, *H2-DMb2*, and *H2-Eb1* (Fig. 3D). Remarkably, all five encode for MHC class II proteins crucial for the activation of CD4^+^ T cells. As illustrated in Fig. 3D, the class II transactivator (*Ciita*) is a major interacting protein responsible for directing MHC II antigen-presentation machinery and is differentially up-regulated in infected susceptible mice (*p* = 3.64 × 10^−5^). Similarly, the invariant chain CD74 involved in the expression and peptide loading of MHC II molecules was also differentially up-regulated in infected susceptible mice (*p* = 1.40 × 10^−6^) (Fig. 3D). MHC II antigen presentation stimulates naïve CD4^+^ T cells to activate major T helper (Th) cell pathways. Indeed, we detected substantially more Tnf-α^+^ Th1 and IL-17A^+^/IL-22^+^ Th17 cells both in frequency and in total numbers in the colonic lamina propria of infected susceptible mice compared to resistant congenic mice (Fig. 5A). Collectively, these data suggest that increased antigen processing and presentation on MHC II molecules in infection-susceptible mice leads to exaggerated CD4^+^ T cell immune activation. GO term enrichment analysis revealed similar patterns (Supplementary Table S4).

#### Functional assessment of the role of CD4^+^ T cells in infection susceptibility

To determine the relationship between the up-regulated CD4^+^ T cell response and susceptibility to infection, susceptible mice were administered the CD4^+^ T-cell-depleting rat anti-mouse CD4 (GK1.5) monoclonal antibody on day 0 and day 2 post-infection with *C*. *rodentium* (Supplementary Fig. S2). Control mice received an equivalent intraperitoneal dose of an isotype-matched antibody. Pathogen loads, body weight loss, *Rspo2* mRNA induction, and crypt hyperplasia were not significantly different between the two groups (Fig. 5B–E), consistent with past studies showing that lymphocytes do not control *C*. *rodentium* colonization^9,38^ or contribute to crypt hyperplasia^39^ at early stages of infection. This firmly places *Rspo2* induction upstream of the observed increase in the CD4^+^ T cell response. Lastly, histopathological scoring was performed to evaluate whether increased CD4^+^ T cell responses contribute to immunopathology in infected susceptible mice. Overall, scores were similar between infected CD4^+^ T-cell-depleted mice and control mice, although a near significant increase in surface epithelial injury was observed in CD4^+^ T-cell-depleted mice (*p* = 0.0606), suggesting that CD4^+^ T cells may be protective in this context (Fig. 5F).

## Discussion

*Citrobacter rodentium* is a mouse-restricted enteric pathogen that causes colitis with marked infiltration of inflammatory cells and a characteristic colonic hyperplasia^1,29^. While most mouse strains suffer relatively mild, self-limiting inflammation following *C*. *rodentium* infection, we have shown that susceptible mouse strains suffer fatal diarrheal disease due to robust *Rspo2* induction during infection^3^. However, the pathways downstream of *Rspo2* and the mechanisms leading to the breakdown of intestinal homeostasis and the development of intestinal dysfunction are still not fully understood. In the present study, we applied an unbiased, global approach through RNA sequencing technology to characterize the shared response to *C*. *rodentium* infection in susceptible and resistant congenic mice, and the *Rspo2*-mediated intestinal dysfunction pathway signature and networks that regulate loss of intestinal homeostasis and disease tolerance. While past studies have performed gene expression profiling of *C*. *rodentium*-infected susceptible and resistant mice using microarrays^13,32^, our experimental design offers two major advantages. First, contrary to comparing the host effect to infection using divergent mouse strains, which have numerous genetic and phenotypic differences, our unique susceptible and resistant congenic strains provide an excellent means to study the biological effects of *Rspo2* independent of the remaining genome. Second, RNA-seq allows for unbiased detection of novel transcripts since it does not require transcript-specific probes like in microarrays.

We first assessed the global shared response to infection in both strains by performing differential expression analysis between the uninfected and infected groups using edgeR and DESeq2. As previously reported, genes involved in ion transport activity and immune responses were highly modulated during infection^13,32^. There was an overall decreased ion transport activity during infection (albeit more so in susceptible mice due to loss of intestinal differentiation) as exemplified by the reduced expression levels of the solute carriers *Slc4a4* and *Slc9a2* in both susceptible and resistant congenic mice. Infection with *C*. *rodentium* provokes a robust immune response characterized by a mixed Th1/Th17 response^29,40,41^. Consistent with existing microarray data of infected resistant C57BL/6 mice^13^, our functional enrichment analysis revealed a similar up-regulation of several immunoregulatory pathways of chemokines and cytokines in our susceptible mice. Although this suggests that the host immune response to infection is not impaired in susceptible mice, whether these immunoregulators are able to properly direct chemotaxis and elicit appropriate inflammatory functions in our mice has not been addressed here.

Other over-represented pathways were involved in changes in metabolism and ECM organization. We observed a striking modulation of key genes involved in the Warburg-like metabolic effect including *Slc16a3*, *Ldha*, and *Ppargc1a* that together lead to decreased pyruvate oxidation. Rapidly dividing cells in the lower colonic crypt utilize oxidative metabolism to a lesser extent than differentiated cells at the luminal surface^15,16^. The modulation of genes involved in mediating the Warburg effect may reflect a metabolic shift in the epithelium due to the expansion of undifferentiated proliferating cells that occurs during *C*. *rodentium* infection. This phenomenon has recently been suggested to contribute to the overgrowth of aerobic *C*. *rodentium* in the colon due to increased oxygenation of the mucosal surface^42^. Another metabolic pathway shown to be enriched during infection was in arachidonic acid metabolism as exemplified by the increase in phospholipases responsible for the release of arachidonic acid from cell membranes. Upon release, arachidonic acid is involved in three major pathways: the cyclooxygenase (Cox) pathway, the epoxygenase pathway, and the lipoxygenase (Alox) pathway^43^. Our RNA-seq dataset revealed decreased expression in *Cox-1* (also known as *Ptgs1*) and no significant modulation of *Cox-2* (also known as *Ptgs2*). Similarly, we observed decreased expression of the majority of epoxygenases including *Cyp2c44*, *Cyp2c55*, *Cyp2c65*, and *Cyp2c68*. In contrast, the observed induction of *Alox12*, albeit higher in susceptible mice, suggests that the lipoxygenase pathway may be the major pathway involved in arachidonic acid metabolism during *C*. *rodentium* infection.

The ECM provides a physical framework for cells and tissues and is essential for various physiological processes including cell adhesion, migration, proliferation, and differentiation. As such, ECM remodeling events are frequently seen in wound healing processes as well as in cancers^21^. We observed a number of genes within our top 20 DEG list that are highly relevant for ECM organization including a profound up-regulation of genes involved in collagen deposition. Collagens provide a scaffold for ECM assembly and its deposition is critical for promoting structural integrity during mucosal wound repair. Indeed, prolyl-4-hydroxylases such as *P4ha1* are essential for pro-collagen biosynthesis while overexpression of *Lox* in dermal wounds has been shown to enhance the mechanical strength of collagen scaffolds through collagen crosslinking^44,45^. Furthermore, we observed an increase in *Pdpn* expression, which is present on lymphatic stromal cells important for the secretion of ECM proteins and the regulation of tissue remodeling. However, we also noted reduced expression of major structural components of the ECM and genes involved in microtubule polymerization. Notably, loss of *Col4a5* and *Col4a6* chains in CRC tissues have been suggested to be related to the observation that cancer cells break through the epithelial basement membrane during the early stages of cancer cell invasion^28^. Collectively, our data suggest that the normal production and organization of the ECM might also be disrupted during *C*. *rodentium* infection.

We next assessed strain-specific gene expression patterns following *C*. *rodentium*. In our previous work, we proposed that the robust induction of *Rspo2* expression in infected susceptible mice leads to pathological activation of Wnt signaling, intestinal epithelial proliferation, and generation of a poorly differentiated colonic epithelium^3^. Our GSEA results comparing infected susceptible mice to resistant congenic mice provide further evidence of a positive correlation between *Rspo2* induction and Wnt targets and stem cell genes while providing evidence of a negative correlation between *Rspo2* induction and genes differentially expressed in the upper colonic crypts, which are enriched for mature differentiated cells. Furthermore, our top 20 DEG lists and GSEA revealed a number of colon cancer-related genes including Wnt target genes that were differentially regulated in infected susceptible mice. Past studies using mouse CRC models have shown that *C*. *rodentium* infection can promote cancer development, but the underlying mechanism behind this effect was not fully demonstrated^46,47^. While our data supports the hypothesis that *Rspo2* induction in inflamed or infected tissue could augment CRC disease, it is difficult to address considering infection-susceptible mice display high mortality following *C*. *rodentium* infection.

Functional enrichment analysis using the KEGG database and GO terms revealed a range of pathways and biological processes that were affected differentially in susceptible versus resistant congenic mice following *C*. *rodentium* infection. Heavily involved in the majority of these pathways were H-2 MHC genes encoding for MHC class II proteins. Naïve CD4^+^ T cells interact directly with MHC II molecules to activate major Th cell subtypes. Indeed, we detected substantially more Tnf-α^+^ Th1 and IL-17A^+^/IL-22^+^ Th17 cells in the colonic lamina propria of infected susceptible mice compared to resistant congenic mice. Our data suggest that the increase in CD4^+^ T cell response is an indirect effect of *Rspo2*, for example, due to loss of epithelial function and in the mucosal barrier, rather than a direct consequence of *Rspo2* on immune cells since we did not observe increases in active β-catenin in immune cells of the colonic lamina propria during infection (data not shown).

Further supporting this hypothesis, we previously reported a pronounced loss of goblet cells as measured by Alcian blue staining in *C*. *rodentium*-infected susceptible mice compared to resistant congenic mice^3^. The intestinal mucus, composed largely of glycosylated *Muc2* mucin, forms a physical barrier to limit the interaction between luminal bacteria and the host epithelium^48^. However, mucus degradation or loss can expose the epithelium to intestinal bacteria, inducing inflammation. Our RNA-seq dataset confirmed a significant down-regulation of *Muc2* expression in infected susceptible mice compared to resistant congenic mice (*p* = 8.62 × 10^−6^). A weaker mucosal barrier due to loss of intestinal differentiation in infected susceptible mice could allow for increased exposure of the epithelium to bacteria, leading to enhanced antigen uptake by antigen-presenting cells and greater CD4^+^ T cell responses. Notably, adhesion of *C*. *rodentium* to intestinal epithelial cells has recently been demonstrated to be a critical cue for Th17 cell induction^49^.

**Figure 6:**
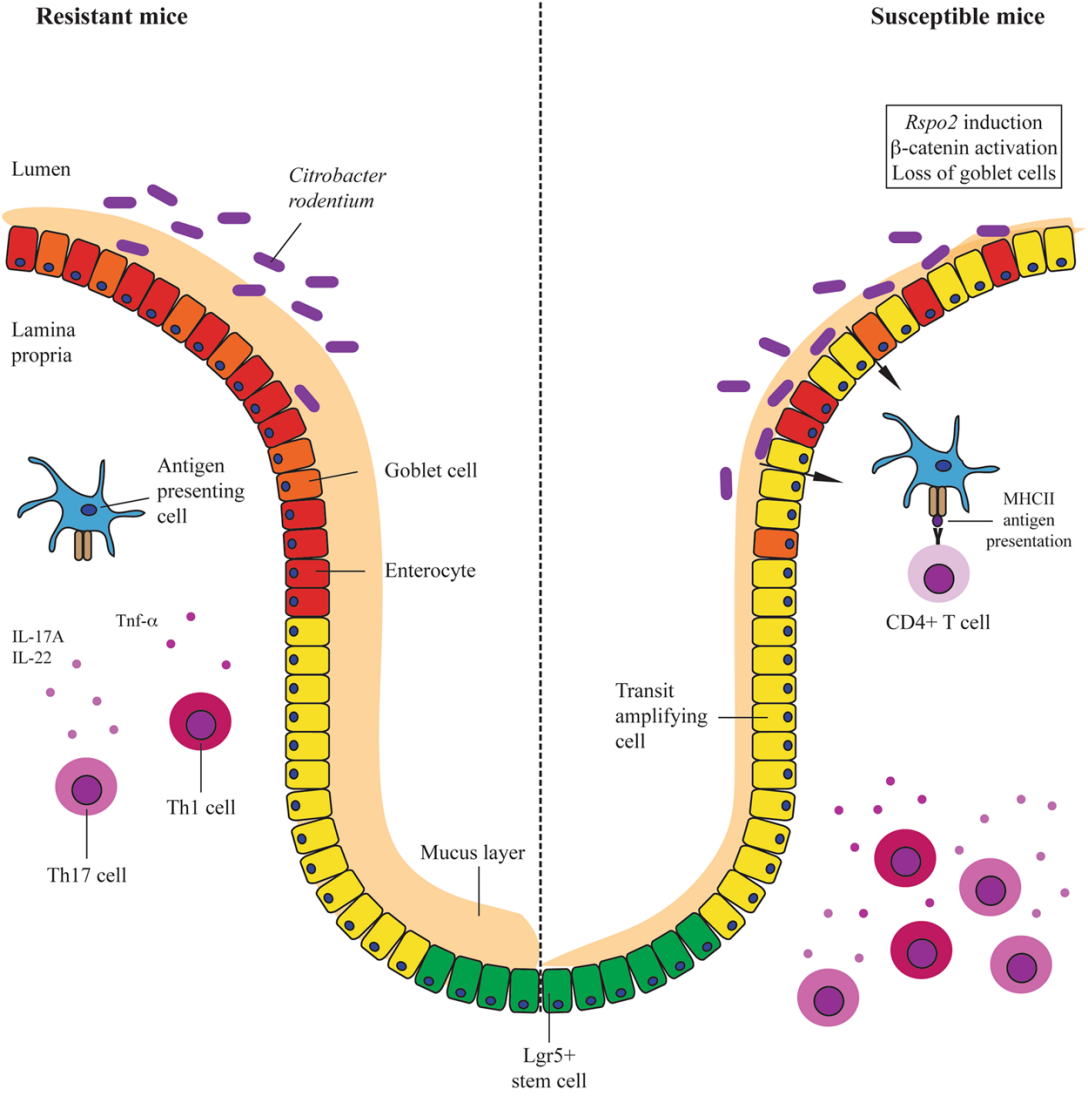
Model for loss of disease tolerance in susceptible mice infected with *C*. *rodentium*. Despite identical bacterial colonization and infection kinetics in susceptible and resistant congenic mice, *Rspo2* induction during infection in susceptible mice leads to pathological activation of Wnt/β-catenin signaling, epithelial proliferation, and generation of a poorly differentiated colonic epithelium. Decreased mucin secretion due to reduced goblet cell numbers allow for increased exposure of the epithelium to bacteria, causing increased bacterial uptake and antigen presentation on MHC class II molecules. The hyperactivation of CD4^+^ T cells result in amplified Tnf-α^+^ Th1 and IL-17A^+^/IL-22^+^ Th17 cell responses.

Mouse models of human CRC have shown that genetic activation of β-catenin can lead to epithelial barrier loss and activation of Th17 cell responses that promote tumour growth^50^. Similarly, infection with the human colonic bacterium enterotoxigenic *Bacteroides fragilis* triggered colitis and tumour development through a Th17-dependent pathway in a mouse model of CRC^51^. Taken together, our data demonstrate that *C*. *rodentium* infection of susceptible inbred mice may mimic CRC pathogenesis through *Rspo2*-mediated signaling: induction of *Rspo2* expression activates β-catenin and induces excessive epithelial proliferation accompanied by a loss of functional cell types including mucin-secreting goblet cells. The subsequent impairment in mucus secretion can lead to increased bacterial-epithelial cell contact and result in hyperactivation of CD4^+^ T cell responses (Fig. 6). We observed no significant contribution of CD4^+^ T cells to immunopathology in susceptible C3Ou mice, in agreement with a previous study showing similar degrees of mucosal hyperplasia and inflammation at 10 days post-infection in wild-type C57BL/6 and in mice lacking mature T and B cells (RAG1^−/−^)^39^. However, the same study showed attenuated colonic pathology in RAG1^−/−^ mice compared to wild-type mice at 18 days post-infection, a time point at which susceptible C3Ou mice have already succumbed to infection.

To summarize, we comprehensively analyzed the global shared response to *C*. *rodentium* infection in susceptible and resistant congenic mice, and examined the *Rspo2*-mediated intestinal dysfunction pathway signature and networks in susceptible mice. Our analysis highlighted changes in host metabolism and tissue remodeling as common responses to infection while providing evidence for a role of antigen processing and presentation of MHC II molecules in disease tolerance as a direct or indirect consequence of *Rspo2* induction in susceptible mice. In conclusion, we identified novel pathways that were not previously associated with *C*. *rodentium* infection and *Rspo2*-mediated signaling, hence providing new insights into the underlying mechanisms regulating intestinal homeostasis versus dysfunction.

## Methods

### Ethics statement

All breeding and experimental procedures were conducted in strict accordance with the Canadian Council of Animal Care and approved by the McGill University Animal Care Committee (permit #5009). Mice were euthanized by CO_2_ asphyxiation and all efforts were made to minimize suffering.

### Mice and *in vivo C. rodentium* infection

C3Ou (Jackson Laboratory, Bar Harbor, ME) and C3Ou.B6-Cri1 congenic mice^6^ carrying an introgressed segment of chromosome 15 from C57BL/6 mice on the C3Ou genomic background were housed in a specific-pathogen free animal facility at McGill University and provided standard mouse chow and water *ad libitum*. For *C*. *rodentium* infections, the *C*. *rodentium* strain DBS100 was grown overnight in 3 ml of LB medium shaking at 37 °C. Five-week-old female mice (three per group) were infected by oral gavage with 0.1 ml of LB medium containing 2–3 × 10^8^ colony-forming units (CFU) of bacteria. Mice were monitored daily and euthanized on day 9 post-infection. Distal colons were harvested and snap frozen in liquid nitrogen for RNA extraction and sequencing.

### RNA sequencing and data analysis

Total RNA of colon tissues from uninfected and infected mice was isolated using TRIzol (Invitrogen) according to the manufacturer’s instructions and a cleanup of all samples was done using the RNeasy Plus Micro Kit (Qiagen). RNA quality was assessed by a Bioanalyzer (Agilent) and sequencing was performed at the McGill University and Genome Quebec Innovation Centre using the Illumina HiSeq. 2000/2500 sequencer to generate 110–187 million 100-bp paired-end reads per library. Trimming was done with the Trimmomatic software^52^ and filtered reads were aligned to the mm10 mouse genome assembly (http://genome.ucsc.edu/cgi-bin/hgGateway?db = mm10) with the combination of the TopHat/Bowtie software^53^. edgeR and DESeq2 Bioconductor packages within the R environment were used to evaluate differential expression, following standard normalisation procedures. Volcano plots and PCA were produced using the ggplot2 package (http://ggplot2.org/) while heatmaps were generated using the pheatmap package (https://cran.r-project.org/web/packages/pheatmap/index.html) within R. Twenty genes found to have baseline differences between the two strains were removed from functional enrichment analysis: *Atp12a*, *BC025446*, *Cyp2w1*, *Frem1*, *Gyk*, *H2-DMa*, *Hkdc1*, *Ighg2b*, *Ighg2c*, *Ighv8-12*, *Ly6d*, *Ly6e*, *Ly6g*, *Per1*, *Prr15*, *Rxfp1*, *Sectm1a*, *Slc36a1*, *St6galnac5*, *Usp2*. DEGs were subject to over-representation analysis using GOseq^54^ and clusterProfiler^55^ Bioconductor packages to determine enriched GO^56^ and KEGG^57^ terms, respectively. NetworkAnalyst^58^ was used to visualize and project DEGs in the context of mouse protein-protein interactome based on the STRING database^59^. In our analysis, a threshold of confidence value 0.900 was used to filter out predicted interactions with lower confidence. For gene set enrichment analysis, non-redundant genes were ranked in order of expression ratios (combination of log_2_ fold change and FDR value) and the ranked gene list was probed with different gene sets as described in the text using the Preranked tool in GSEA (http://software.broadinstitute.org/gsea/index.jsp). The KEGG-derived CRC gene set is available in the MSigDB C2 collection in GSEA. Duplicate probes were collapsed to the highest value. Default parameters were applied: 1000 permutations and a weighted enrichment statistic.

### Flow cytometry analysis

Colonic lamina propria cells from mice were isolated using a modified version of a previously described method^60^. In brief, colons were harvested, cut open longitudinally into 1 cm pieces, and washed in calcium- and magnesium-free HBSS (Gibco) supplemented with 2% heat-inactivated FCS (Wisent) and 15 mM HEPES (Gibco). The resulting tissue pieces were washed in calcium- and magnesium-free HBSS supplemented with 2% FCS, 15 mM HEPES, and 5 mM EDTA to remove epithelial cells. Tissue pieces were then incubated in RPMI-1640 (Sigma) supplemented with 10% FCS, 15 mM HEPES, 160 μg/ml collagenase IV (Sigma), and 40 μg/ml DNAse I (Roche) for 40 min at 37 °C. The cell suspension was filtered through a 70 μm cell strainer (Sigma) before proceeding with cell stimulation. For cytokine detection, cells were stimulated with 50 ng/ml PMA and 500 ng/ml ionomycin in the presence of the Protein Transport Inhibitor Cocktail from eBioscience for 3 hr at 37 °C. Stimulated cells were stained with viability dye (Life Technologies) and with fluorescently labeled surface antibodies CD45 (30-F11), CD4 (RM4-5), and TCRβ(H57-597) from eBioscience. Cells were then fixed and permeabilized with a fixation and permeabilization kit from eBioscience according to the manufacturer’s instructions, and intracellularly stained with fluorescently labeled Tnf-α (MP6-XT22), IL-17A (eBio17B7), and IL-22 (1H8PWSR) antibodies from eBioscience. Cells were acquired on the FACSCanto II cytometer (BD Biosciences) and data was analyzed using FlowJo software (Tree Star).

### *In vivo* CD4^+^ T cell depletion

CD4^+^ T-cell-depleting monoclonal antibody (GK1.5) and rat IgG2b isotype-matched control antibody (LTF-2) were obtained from Bio X Cell. Mice were administered 200 μg of either GK1.5 or LTF-2 intraperitoneally on the day of oral infection with *C*. *rodentium* and again on day 2 post-infection. The efficacy of depletion in infected mice was monitored by flow-cytometric analysis of blood and of the colonic lamina propria using the noncompeting anti-CD4 RM4-4 antibody from eBioscience (Supplementary Fig. S2). Body weight was measured on a daily basis until mice were euthanized on day 9 post-infection. Distal colons were harvested for CFU counts, RNA extraction, and histology.

### CFU count and qRT-PCR

For the *in vivo* CD4^+^ T cell depletion experiment, stool samples were collected on day 3 post-infection and distal colonic tissues on day 9 post-infection. The number of viable bacteria per gram of stool and colonic tissue was determined by serial dilution plating onto MacConkey agar. Total RNA from colons were isolated using TRIzol (Invitrogen) according to the manufacturer’s instructions. The purity of RNA was assessed by a spectrophotometer and complementary DNA was synthesized from 1 μg of RNA with RevertAid Reverse Transcriptase (Thermo Scientific) and random primers (Invitrogen) using an Eppendorf PCR thermal cycler. *Rspo2* expression levels were measured using TaqMan Gene Expression Assay (Applied Biosystems) on the Applied Biosystems StepOnePlus Real-Time PCR system. Analysis was performed according to the comparative C^T^ method using *Hprt* as the housekeeping gene.

### Histopathology scoring and crypt length measurement

Colon sections of infected CD4^+^ T-cell-depleted mice and control mice were fixed in 10% buffered formalin, paraffin-embedded, sectioned at 5 μm, and stained for hematoxylin and eosin (H&E). H&E sections were scored by an expert pathologist in a blinded manner: inflammatory cell infiltration (where 0 = occasional resident inflammatory cells in the lamina propria, 1 = minimal increase in inflammatory cells, 2 = mild increase in inflammatory cells, 3 = moderate increase in inflammatory cells, 4 = marked increase in inflammatory cells), inflammatory cell localization or depth (0 = no significant inflammatory infiltration, 1 = infiltration localized to the lamina propria, 2 = infiltration extended significantly into the submucosa, 3 = infiltration extended significantly into the muscularis, 4 = infiltration extended significantly to the serosa/mesentery), submucosal edema (0 = no edema, 1 = mild edema, few areas, 2 = mild edema in frequent areas or moderate edema in few areas, 3 = moderate edema in frequent or extensive areas, 4 = marked edema, frequent to diffuse), bacterial colonization (0 = no significant number of bacteria adhered to the mucosal surface, 1 = presence of rare to occasional colonization of epithelial surface with extension into no or few glands, 2 = abundant colonization of the epithelial surface with extension into occasional glands, 3 = abundant colonization of the epithelial surface extending to numerous glands, 4 = abundant colonization of the epithelial surface and invasion into the lamina propia or submucosa), surface epithelial injury (0 = normal surface epithelium, 1 = rare to occasional areas of epithelial flattening, degeneration or exfoliation, 2 = frequent areas of epithelial flattening, degeneration, or exfoliation, 3 = frequent areas of epithelial flattening, degeneration, or exfoliation with rare areas of epithelial erosion/ulceration, 4 = frequent or extensive areas of epithelial ulceration), mucosal necrosis (0 = none, 1 = rare, small foci of mucosal necrosis, 2 = occasional small foci of mucosal necrosis, 3 = frequent small foci of mucosal necrosis or rare wide foci, 4 = extensive areas of mucosal necrosis), goblet cell/enterocyte ratio decrease, (0 = normal, 1 = decrease in goblet cell proportion affecting few glands, 2 = decrease in goblet cell proportion affecting occasional glands, 3 = decrease in goblet cell proportion affecting frequent glands, 4 = decrease in goblet cell proportion affecting most or all of the tissue), and gland loss (0 = normal density of glands, 1 = rare, small foci of gland loss over small areas, 2 = occasional small foci of gland loss, 3 = frequent small foci of gland loss or rare wide foci, 4 = extensive areas of gland loss). The maximum combined score that could be obtained with this system was 32. Lastly, crypt lengths were quantified by measuring the average depth of approximately 15 well-oriented colonic crypts for each mouse.

### Statistical analysis

Data analyses were performed using GraphPad Prism v6.0 software. Statistical comparison between groups was carried out using tests described in the figure legends. A *p* < 0.05 was considered statistically significant.

### Data Availability

The RNA-seq dataset from this study has been deposited in the Gene Expression Omnibus (GEO) database under the accession number GSE100546.

## Electronic supplementary material

Supplementary Information
