# Supplementary material for: Loss of disease tolerance during Citrobacter rodentium infection is associated with impaired epithelial differentiation and hyperactivation of T cell responses
Source: Sci Rep. 2018 Jan 16;8:847. doi: 10.1038/s41598-017-17386-y (PMC5770458; doi:10.1038/s41598-017-17386-y)
Supplement: Supplementary file 1 — Supplementary Information [file 41598_2017_17386_MOESM1_ESM.pdf]

Supplementary Information

Loss of disease tolerance during *Citrobacter rodentium* infection is associated with impaired epithelial differentiation and hyperactivation of T cell responses

Eugene Kang[1,2], Guangyan Zhou[3], Mitra Yousefi[1,2], Romain Cayrol[4], Jianguo Xia[1,3,5], Samantha Gruenheid[1,2]*

Supplementary Figure

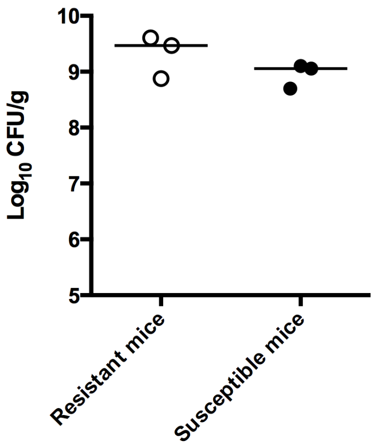

Supplementary Figure S1. Bacterial loads are identical in susceptible and resistant congenic mice at 9 days post-infection. Fecal pellets were collected at day 9 post-infection and bacterial counts were determined by plating of serial dilutions on MacConkey agar (n=3). Bacterial counts were log10 transformed. Bars indicate median values.

Supplementary Figure

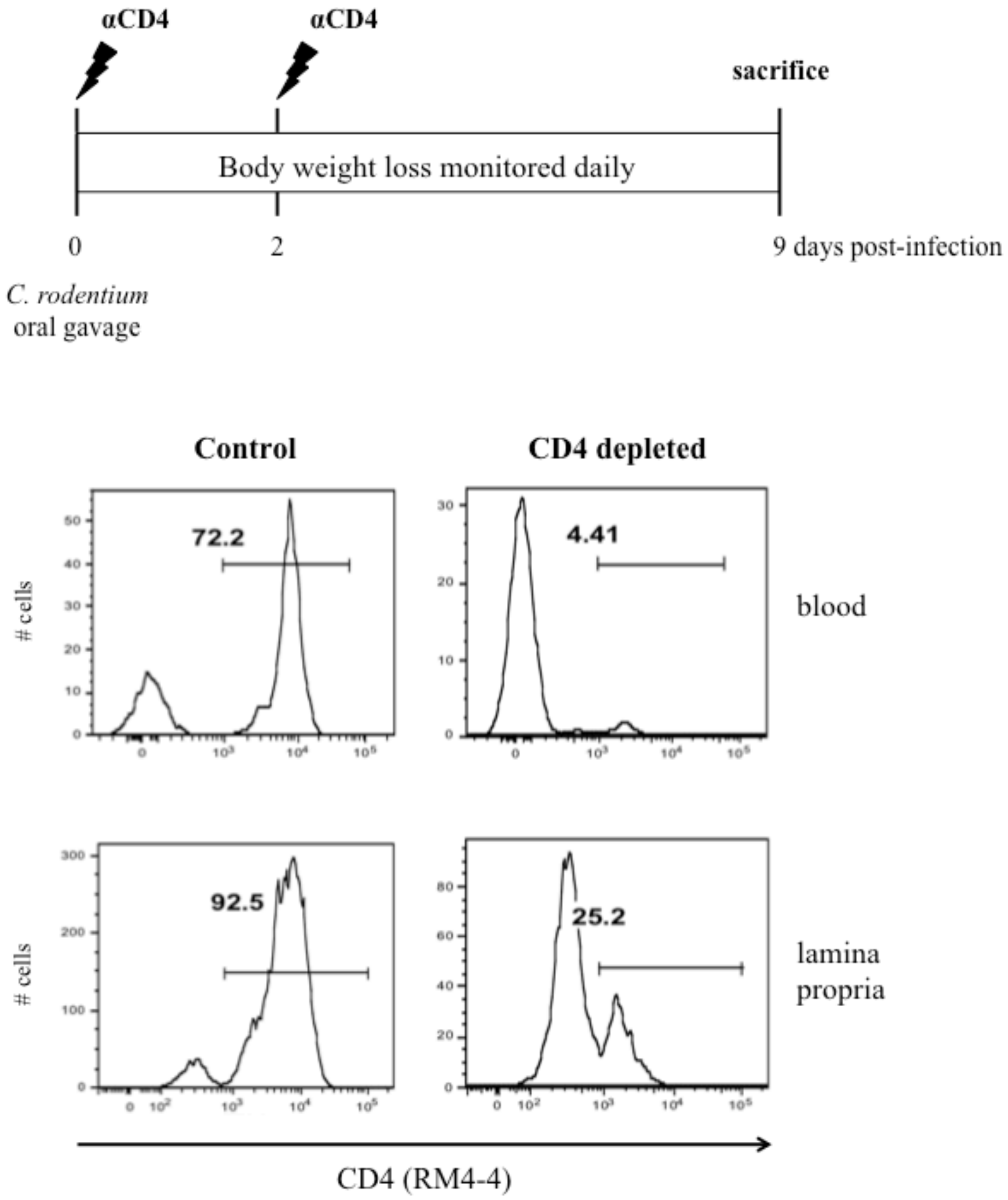

Supplementary Figure S2. Schematic diagram of the *in vivo* $CD4^+$ T cell depletion and efficacy of depletion in the blood and colonic lamina propria as measured by flow cytometry. Mice (n=6 per group) were administered 200 μg of either GK1.5 or control LTF-2 intraperitoneally on the day of oral infection with *C. rodentium* and again on day 2 post-infection before sacrifice on day 9 post-infection. Blood was collected via tail incisions just prior to sacrifice while colonic lamina propria cells were harvested upon sacrifice as described in the main article. Cells were gated on live, singlet $CD45^+$ and $TCR\beta^+$ and subsequently on CD4 (noncompeting clone RM4-4). Representative histograms of the depletion are shown for blood and colonic lamina propria.

Supplementary Table

ID	P-value	Description
mmu04657	1.48E-09	IL-17 signaling pathway
mmu04060	2.91E-09	Cytokine-cytokine receptor interaction
mmu04080	4.25E-08	Neuroactive ligand-receptor interaction
mmu04726	7.46E-06	Serotonergic synapse
mmu00140	2.36E-03	Steroid hormone biosynthesis
mmu00591	2.36E-03	Linoleic acid metabolism
mmu00590	2.64E-03	Arachidonic acid metabolism
mmu04972	3.67E-03	Pancreatic secretion
mmu04020	9.28E-03	Calcium signaling pathway
mmu04668	1.50E-02	TNF signaling pathway

Supplementary Table S1. Top ten statistically over-represented KEGG pathway terms in susceptible and resistant congenic mice in response to *C. rodentium* infection.

Supplementary Table

GO ID	P-value	Description
GO:0007267	7.97E-13	cell-cell signaling
GO:0007610	2.02E-12	behavior
GO:0006811	2.11E-12	ion transport
GO:0007186	4.99E-12	G-protein coupled receptor signaling pathway
GO:0050877	7.35E-11	neurological system process
GO:0042221	9.21E-10	response to chemical
GO:0099536	2.80E-09	synaptic signaling
GO:0019369	5.29E-09	arachidonic acid metabolic process
GO:0034097	6.70E-09	response to cytokine
GO:0006952	9.81E-09	defence response

Supplementary Table S2. Top over-represented GO biological processes in susceptible and resistant congenic mice in response to *C. rodentium* infection.

Supplementary Table

ID	P-value	Description
mmu05310	6.58E-05	Asthma
mmu04514	8.29E-05	Cell adhesion molecules (CAMs)
mmu05321	8.24E-04	Inflammatory bowel disease (IBD)
mmu04672	8.24E-04	Intestinal immune network for IgA production
mmu04659	1.05E-03	Th17 cell differentiation
mmu04658	1.46E-03	Th1 and Th2 cell differentiation
mmu05150	1.57E-03	Staphylococcus aureus infection
mmu04612	1.57E-03	Antigen processing and presentation
mmu04640	1.96E-03	Hematopoietic cell lineage
mmu05323	3.78E-03	Rheumatoid arthritis

Supplementary Table S3. Top ten statistically over-represented KEGG pathway terms in infected susceptible mice compared to resistant congenic mice.

Supplementary Table

GO ID	P-value	Description
GO:0019886	1.82E-10	antigen processing and presentation of exogenous peptide antigen via MHC class II
GO:0009605	6.83E-09	response to external stimulus
GO:0010817	1.51E-08	regulation of hormone levels
GO:0051239	2.49E-07	regulation of multicellular organismal process
GO:0006954	2.90E-07	inflammatory response
GO:0007610	6.64E-07	behavior
GO:0009611	8.73E-07	response to wounding
GO:0042127	9.38E-07	regulation of cell proliferation
GO:0006935	1.36E-06	chemotaxis
GO:0007155	5.86E-06	cell adhesion

Supplementary Table S4. Top over-represented GO biological processes in infected susceptible mice compared to resistant congenic mice.

Supplementary Table

Top 20 Up-regulated Genes			Top 20 Down-regulated Genes		
**Symbol**	**P-value**	**Name**	**Symbol**	**P-value**	**Name**
Neto2	0	neuropilin and tolloid-like 2	Gm12551	2.25E-135	predicted gene 12551
Tgm2	0	transglutaminase 2, C polypeptide	Slc4a4	1.03E-130	solute carrier family 4, member 4
Pdpn	0	podoplanin	Sycn	7.76E-127	syncollin
S100a11	0	S100 calcium binding protein A11 (calgizzarin)	Syn2	8.45E-120	synapsin II
Mfsd2a	6.63E-298	major facilitator superfamily domain containing 2A	Edil3	9.14E-111	EGF-like repeats and discoidin I-like domains 3
Hsp90aa1	1.25E-279	heat shock protein 90, alpha (cytosolic), class A member 1	Trpv3	2.91E-106	transient receptor potential cation channel, subfamily V, member 3
Atp1b2	1.54E-257	ATPase, Na+/K+ transporting, beta 2 polypeptide	Iyd	5.60E-105	iodotyrosine deiodinase
Cebpb	1.77E-179	CCAAT/enhancer binding protein (C/EBP), beta	Tppp	5.75E-104	tubulin polymerization promoting protein
P4ha1	4.88E-167	procollagen-proline, proline 4-hydroxylase, alpha 1	Fam189a2	3.23E-102	family with sequence similarity 189, member A2
Uck2	1.78E-160	uridine-cytidine kinase 2	Col6a4	5.90E-102	collagen, type VI, alpha 4
Abca1	4.46E-154	ATP-binding cassette, sub-family A (ABC1), member 1	Tppp3	4.37E-98	tubulin polymerization-promoting protein family member 3
AI747448	1.93E-148	expressed sequence AI747448	Hapln1	4.58E-97	hyaluronan and proteoglycan link protein 1
Tnfrsf23	5.22E-146	tumor necrosis factor receptor superfamily, member 23	Col4a6	1.98E-88	collagen, type IV, alpha 6
Slc25a37	8.92E-135	solute carrier family 25, member 37	Insrr	3.94E-84	insulin receptor-related receptor
Prss22	1.00E-127	protease, serine, 22	Itih2	3.18E-80	inter-alpha trypsin inhibitor, heavy chain 2
Ltf	1.23E-118	lactotransferrin	Esrrg	8.16E-80	estrogen-related receptor gamma
Lox	4.06E-117	lysyl oxidase	Fmo5	2.53E-78	flavin containing monooxygenase 5
Dhrs9	2.87E-116	dehydrogenase/reductase (SDR family) member 9	Mapk15	7.55E-78	mitogen-activated protein kinase 15
Duoxa2	3.13E-113	dual oxidase maturation factor 2	Cybrd1	7.99E-78	cytochrome b reductase 1
Hsd11b1	7.75E-111	hydroxysteroid 11-beta dehydrogenase 1	Naaladl1	1.97E-74	N-acetylated alpha-linked acidic dipeptidase-like 1

Supplementary Table S5. Top 20 up-regulated and down-regulated differentially expressed genes in infected susceptible and resistant congenic mice based on DESeq2.

Supplementary Table

Top 20 Up-regulated Genes			Top 20 Down-regulated Genes		
**Symbol**	**P-value**	**Name**	**Symbol**	**P-value**	**Name**
Gm830	8.73E-41	predicted gene 830	Slc26a3	4.23E-60	solute carrier family 26, member 3
Rspo2	3.50E-36	R-spondin 2 homolog (Xenopus laevis)	Tmigd1	3.08E-33	transmembrane and immunoglobulin domain containing 1
Rnf43	2.84E-20	ring finger protein 43	Agr2	5.10E-19	anterior gradient 2
Mettl7a3	6.09E-18	methyltransferase like 7A3	Car4	2.87E-17	carbonic anhydrase 4
Pvrl4	4.97E-16	poliovirus receptor-related 4	Slc51b	2.87E-17	solute carrier family 51 beta subunit
Alox12	9.38E-16	arachidonate 12-lipoxygenase	Ankdd1b	2.96E-16	ankyrin repeat and death domain containing 1B
Sptbn2	6.45E-14	spectrin beta, non-erythrocytic 2	Sult1c2	5.08E-16	sulfotransferase family, cytosolic, 1C, member 2
Mycl1	1.22E-13	v-myc myelocytomatosis viral oncogene homolog 1, lung carcinoma derived	Mep1b	3.37E-15	meprin 1 beta
Chodl	2.46E-13	chondrolectin	Tmem181c-ps	1.05E-12	transmembrane protein 181C, pseudogene
Spns2	2.93E-13	spinster homolog 2	Tat	1.39E-11	tyrosine aminotransferase
Tmprss13	2.71E-12	transmembrane protease, serine 13	Clca6	1.39E-11	chloride channel calcium activated 6
Ephb3	1.57E-11	Eph receptor B3	Kif5c	1.56E-11	kinesin family member 5C
Axin2	1.03E-10	axin2	6430548 M08Rik	1.70E-11	RIKEN cDNA 6430548M08 gene
Jun	3.15E-10	Jun oncogene	Ildr1	1.30E-10	immunoglobulin-like domain containing receptor 1
Wfikkn2	7.03E-10	WAP, follistatin/kazal, immunoglobulin, kunitz and netrin domain containing 2	Zg16	1.94E-10	zymogen granule protein 16
Lrrc66	1.31E-09	leucine rich repeat containing 66	Nefl	2.07E-10	neurofilament, light polypeptide
Trim29	5.87E-09	tripartite motif-containing 29	Kcnk6	2.90E-10	potassium inwardly-rectifying channel, subfamily K, member 6
Foxq1	9.97E-09	forkhead box Q1	Sgk2	3.80E-10	serum/glucocorticoid regulated kinase 2
Tmem132c	1.18E-08	transmembrane protein 132C	Scgn	2.48E-09	secretagogin, EF-hand calcium binding protein
Nfe2l3	1.43E-08	nuclear factor, erythroid derived 2, like 3	Hes2	2.54E-09	hairy and enhancer of split 2 (Drosophila)

Supplementary Table S6. Top 20 up-regulated and down-regulated differentially expressed genes in infected susceptible mice compared to infected resistant congenic mice based on DESeq2.
